# Isotemporal substitution analysis of time between sleep, sedentary behavior, and physical activity on depressive symptoms in older adults: a cross-sectional study

**DOI:** 10.1590/1516-3180.2023.0144.R2.04122023

**Published:** 2024-03-18

**Authors:** Joilson Meneguci, Lucas Lima Galvão, Sheilla Tribess, Cíntia Aparecida Garcia Meneguci, Jair Sindra Virtuoso

**Affiliations:** I PhD. Physical Education Professional, Postgraduate Program in Physical Education, Clinical Hospital, Universidade Federal do Triângulo Mineiro (UFTM), Uberaba (MG), Brasil.; II MSc. Physical Education Professional, PhD Student, Postgraduate Program in Physical Education, Universidade Federal do Espírito Santo (UFES), Vitória (ES), Brasil.; III PhD. Physical Education Professional, Associate Professor, Postgraduate Program in Physical Education, Department of Sport Sciences, Universidade Federal do Triângulo Mineiro (UFTM), Uberaba (MG), Brasil.; IV PhD. Physiotherapist, Clinical Hospital (HC), Universidade Federal do Triângulo Mineiro (UFTM), Uberaba (MG), Brasil. https://orcid.org/; V PhD. Physical Education Professional, Associate Professor, Postgraduate Program in Physical Education, Department of Sport Sciences, Universidade Federal do Triângulo Mineiro (UFTM), Uberaba (MG), Brasil.

**Keywords:** Public health, Aging, Health behavior, Statistical Models, Epidemiology, Sedentary behavior, Sitting time, Elderly, Isotemporal substitution model, Physical exercise

## Abstract

**BACKGROUND:**

Compared to young individuals, older adults participate more in sedentary behavior (SB) and less in physical activity (PA). These behaviors are associated with numerous adverse health factors.

**OBJECTIVE:**

The purpose of the study was to examine the hypothetical effects of substituting time spent sleeping, performing SB, and performing moderate-to-vigorous physical activity (MVPA) on depressive symptomatology in older adults.

**DESIGN AND SETTING:**

An analytical cross-sectional study employing exploratory survey methods was conducted in the city of Alcobaça in the state of Bahia, Brazil

**METHODS:**

The study included 473 older adults who answered a structured questionnaire during an interview. Exposure time to SB and PA level were assessed using the International Physical Activity Questionnaire, and depressive symptoms were analyzed using the short version of the Geriatric Depression Scale. An isotemporal replacement model was used to evaluate the effects of different SB sessions on depressive symptomatology.

**RESULTS:**

An increase in the risk of depressive symptoms was observed when MVPA and sleep time were substituted for the same SB time at all times tested, with maximum values of 40% and 20%, respectively. Opposite substitution of MVPA and sleep time increments reduced the risk of depressive symptomatology by 28% and 17%, respectively.

**CONCLUSIONS:**

The results of the present study indicate that replacing SB with the same amount of sleep or MVPA may reduce depressive symptoms. The longer the reallocation time, the greater are the benefits.

## INTRODUCTION

Depression is considered one of the most prevalent mental disorders in many countries.^1^ It frequently occurs in older adults and results from the interaction of several factors, including genetic factors, cognitive deficits, and disturbing situations.^2^

Depression presents a major economic burden, as it ranks 15th among conditions with the highest healthcare costs,^3^ causes a reduced functional capacity to perform basic activities of daily living, decreases quality of life, and is associated with increased use of health services, hospitalization, and mortality.^4^ ^,^ ^5^ ^,^ ^6^

Specifically, in older adults, previous studies have noted that several factors contribute to the disease.^2^ ^,^ ^7^ Among behavioral factors, a meta-analysis showed that people with depression are less physically active and engage in higher levels of sedentary behavior (SB).^8^ Sleep time has also been shown to be associated with depression, with both short (≤ 6 hours) and long durations (> 9 hours) significantly and more strongly associated with depression than sleep durations between 7 and 9 hours.^9^

Although there is consensus in the literature that intervention strategies based on increased regular physical activity (PA) are effective in reducing depressive symptoms,^10^ it is necessary to consider other behaviors during the day. During a 24-hour period, different behaviors may be adopted: sleep, SB, and PA (light, moderate, and vigorous).^11^ In addition, the interaction of behaviors (sleep, SB, and PA) over the course of 24 hours is directly related to an individual’s health.^12^

The recommended sleep time for older adults is 7-8 hours per day, and the recommended amount of moderate-to-vigorous physical activity (MVPA) is 30 minutes/day.^13^ ^,^ ^14^ Regarding SB, although there is not yet a recommended time per day, its reduction is important for individuals to maintain an active lifestyle, and increases in the time spent in light-intensity activities have been suggested.^15^ ^,^ ^16^

Statistical isotemporal substitution modelling was used to assess the hypothetical effects of the replacement of time spent on activities on health conditions. Thus, hypothetical isotemporal replacement models have gained prominence in the literature and have been applied to assess the reallocation of a given time spent on one activity to the same time spent on another.^17^

The analysis of isotemporal substitution has been applied in several studies in different populations, as highlighted in a recent systematic review.^18^ For example, in a longitudinal study performed in older adults, it was found that the 60-minute replacement of sitting time for standing, walking, MVPA, and sleep for individuals sleeping ≤ 7 hours/day reduced the risk for mortality.^19^

Previous studies have estimated the impact of isotemporal substitution on depressive symptoms, and positive effects were found when 60 minutes of television time was reallocated to walking at an average speed^20^ and 60 minutes of SB time was reallocated to vigorous PA.^21^ Specifically, in older adults, studies have reported the benefits of replacing time spent in SB with time spent in PA on depressive symptoms,^22^ ^,^ ^23^ and the benefits of the replacement of only 30 minutes/day, not including a replacement with sleep, was verified. In addition, these studies were conducted in developed countries.

## OBJECTIVE

This study aimed to examine the hypothetical effects of substituting time spent sleeping, engaged in SB, and performing MVPA on depressive symptomatology in older adults. We hypothesized that the substitution of time spent engaged in MVPA with the same amount of time spent in SB could increase the likelihood of developing depressive symptomatology and that inverse substitution when replacing SB with MVPA reduces the chances of developing depressive symptomatology.

## METHODS

### Study sample

A cross-sectional population-based epidemiological survey entitled the Longitudinal Study of the Elderly Health of Alcobaça (ELSIA Project) was conducted with individuals aged ≥ 60 years who were registered with the Family Health Strategy of the Health System of the Brazilian government in the municipality of Alcobaça, Brazil. The exclusion criteria were the presence of cognitive impairment according to the Mini-Mental State Examination,^24^ inability to ambulate even with the assistance of a cane or walker, severe difficulty in visual and auditory acuity according to the interviewer’s perception, wheelchair dependence; and severe sequelae of a cerebrovascular accident with a localized loss of strength.

Among the 743 older adults registered in the Family Health Strategy, 54 refused to participate in the survey, 58 were excluded because they did not meet the inclusion criteria, and 158 could not be located. Thus, the analysis included 473 participants, with data collected from July to October 2015.

Data were collected at an older adult residence by a team of students and trained health professionals. Participants responded to a structured questionnaire during a face-to-face interview. The research protocols were evaluated and approved by the Research Ethics Committee of the Universidade Federal do Triangulo Mineiro (Ordinance No. 966.983/2015; February 25, 2015). All participants provided written informed consent before participation.

### Outcome measures: depressive symptoms

Depressive symptoms were assessed using the Brazilian short-form version of the Geriatric Depression Scale (GDS-SF; Spearman’s rho test-retest reliability = 0.86).^25^ The GDS-SF scale consists of 15 “yes or no” questions. The final score ranges from 0 to 15 points; a score of 6-10 points suggests mild to moderate depression, and a score of 11-15 points suggests serious or severe depression. In this study, the presence of depressive symptoms in older adults was defined as a score of ≥ 6 points.

### Outcome measures: sleep, sedentary behavior, and physical activity

Time spent sleeping at night was measured using a single question, which was part of the Brazilian Portuguese version of the Pittsburgh Sleep Quality Index: “How many hours of actual sleep do you get at night?”^26^

The time in SB and physical activity was determined using the long version of the International Physical Activity Questionnaire (IPAQ), which has been validated for the Brazilian older adult population (Kappa coefficient = 0.27 for women and 0.24 for men; Spearman’s rho test-retest reliability = 0.78 for women and 0.95 for men).^27^ ^,^ ^28^ The participants were asked to report the time spent sitting during a typical weekday and weekend day and the time spent performing moderate- to vigorous-intensity physical activity in a standard week in four domains: work, transportation, domestic activities, and leisure activities.

The time spent engaged in SB during a typical day was calculated as [(time spent sitting during a typical weekday × 5 + time spent sitting during a typical weekend day × 2)/7]. The total time spent in moderate-to-vigorous-intensity physical activity (MVPA) per day was determined using the following formula: time spent in MVPA/day = [total minutes in moderate-intensity physical activity/week + (total minutes in vigorous-intensity physical activity/week × 2)]/7.^29^

### Covariates

Demographic variables included sex (male or female), age (years), and marital status (single/divorced, married, or widowed). These variables were self-reported by participants.

### Statistical analysis

Data were entered twice using Epidata software, version 3.1b (EpiData Association, Odense, Denmark). Statistical analyses were performed using Statistical Package for the Social Sciences version 21.0 (SPSS Inc., Chicago, IL, USA).

To compare the participants’ characteristics according to depressive symptoms, t-tests or chi-square tests were used. To verify the hypothetical effects of reallocation of time spent on sleep, SB, and moderate and vigorous activities on depressive symptoms, the isotemporal substitution approach was used.^17^ The isotemporal substitution analyses were performed by estimating the prevalence ratio (PR) with the respective 95% confidence interval (CI) using Poisson regression with robust variance. Isotemporal substitution models were performed for 5, 10, 15, 20, 25, 30, 35, 40, 45, 50, 55, and 60 minutes/day spent on sleep, SB, and MVPA and depressive symptoms. All models were adjusted for sex, age, and marital status, and the level of statistical significance was set at P < 0.05.

## RESULTS

Of the 473 participants, 62.6% (n = 296) were women, and 46.0% (n = 217) were married. The average age of the participants was 70.2 (± 8.2) years, with a range of 60 to 97 years. On average, the participants spent 436.14 (± 105.94) minutes per day sleeping, 433.68 (± 162.45) minutes engaged in SB, and 52.12 (± 82.08) minutes performing MVPA.

Table 1 presents the participants’ characteristics based on the presence of depressive symptoms. Older adults who were female, were widowers, had longer SB, or had lower MVPA had depressive symptoms (Table 1).

According to the isotemporal substitution models, more time spent sleeping or performing MVPA and less time spent engaged in SB reduced the likelihood of depressive symptoms (P < 0.05). When more time was substituted, the protective effect was greater, and reallocation to MVPA resulted in a greater reduction in depressive symptoms (Table 2).

**Table 1.** Characteristics of the participants by the presence of depressive symptoms

	Absence of depressive symptoms	Presence of depressive symptoms	P
Sex, n (%)			0.041
Male	163 (39.1)	14 (25.0)	
Female	254 (60.9)	42 (75.0)	
Marital status, n (%)			0.004
Single / divorced	110 (26.4)	15 (26.8)	
Married	201 (48.3)	16 (28.6)	
Widowed	105 (25.2)	25 (44.6)	
Age (years)	70.01 ± 8.15	72.00 ± 8.89	0.091
Sleep (min/day)	438.31 ± 103.24	416.50 ± 116.42	0.145
Sedentary behavior (min/day)	424.43 ± 159.49	498.74 ± 162.29	0.001
MVPA (min/day)	54.82 ± 84.62	31.01 ± 40.22	0.001

MVPA = moderate to vigorous physical activity.

**Table 2.** Isotemporal substitution models for depressive symptoms

Isotemporal models	Sleep	SB	MVPA
	PR (95%CI)	PR (95%CI)	PR (95%CI)
*5 minutes/day*			
Replace sleep	Dropped	1.01 (1.01-1.03)*	0.99 (0.96-1.01)
Replace SB	0.98 (0.97-0.99)*	Dropped	0.97 (0.95-0.99)*
Replace MVPA	1.01(0.99-1.04)	1.03 (1.01-1.06)*	Dropped
*10 minutes/day*			
Replace sleep	Dropped	1.03 (1.01-1.06)*	0.97 (0.92-1.03)
Replace SB	0.97 (0.95-0.99)*	Dropped	0.94 (0.89-0.99)*
Replace MVPA	1.02 (0.97-1.08)	1.06 (1.01-1.11)*	Dropped
*15 minutes/day*			
Replace sleep	Dropped	1.05 (1.01-1.08)*	0.96 (0.89-1.04)
Replace SB	0.95 (0.92-0.99)*	Dropped	0.92 (0.85-0.99)*
Replace MVPA	1.04 (0.96-1.13)	1.08 (1.01-1.18)*	Dropped
*20 minutes/day*			
Replace sleep	Dropped	1.06 (1.02-1.11)*	0.95 (0.85-1.06)
Replace SB	0.94 (0.90-0.98)*	Dropped	0.89 (0.80-0.99)*
Replace MVPA	1.05 (0.94-1.17)	1.12 (0.01-1.24)*	Dropped
*25 minutes/day*			
Replace sleep	Dropped	1.08 (1.02-1.14)*	0.94 (0.82-1.08)
Replace SB	0.92 (0.88-0.98)*	Dropped	0.87 (0.76-0.99)*
Replace MVPA	1.06 (0.93-1.22)	1.15 (1.01-1.31)*	Dropped
*30 minutes/day*			
Replace sleep	Dropped	1.10 (1.03-1.17)*	0.93 (0.79-1.09)
Replace SB	0.91 (0.85-0.97)*	Dropped	0.85 (0.72-0.99)*
Replace MVPA	1.08 (0.92-1.27)	1.18 (1.01-1.38)*	Dropped
*35 minutes/day*			
Replace sleep	Dropped	1.11 (1.03-1.20)*	0.92 (0.76-1.11)
Replace SB	0.89 (0.83-0.97)*	Dropped	0.82 (0.68-0.98)*
Replace MVPA	1.09 (0.90-1.32)	1.22 (1.01-1.46)*	Dropped
*40 minutes/day*			
Replace sleep	Dropped	1.13 (1.04-1.24)*	0.90 (0.73-1.12)
Replace SB	0.88 (0.81-0.97)*	Dropped	0.80 (0.65-0.98)*
Replace MVPA	1.10 (0.89-1.37)	1.25 (1.01-1.54)*	Dropped
*45 minutes/day*			
Replace sleep	Dropped	1.15 (1.04-1.27)*	0.89 (0.70-1.14)
Replace SB	0.87 (0.79-0.96)*	Dropped	0.77 (0.61-0.98)*
Replace MVPA	1.12 (0.87-1.43)	1.28 (1.01-1.63)*	Dropped
*50 minutes/day*			
Replace sleep	Dropped	1.17 (1.04-1.30)*	0.88 (0.67-1.16)
Replace SB	0.86 (0.77-0.96)*	Dropped	0.75 (0.58-0.98)*
Replace MVPA	1.13 (0.86-1.48)	1.32 (1.02-1.72)*	Dropped
*55 minutes/day*			
Replace sleep	Dropped	1.18 (1.05-1.34)*	0.87 (0.65-1.17)
Replace SB	0.84 (0.75-0.95)*	Dropped	0.74 (0.55-0.98)*
Replace MVPA	1.15 (0.85-1.55)	1.36 (1.02-1.82)*	Dropped
*60 minutes/day*			
Replace sleep	Dropped	1.20 (1.06-1.37)*	0.86 (0.62-1.19)
Replace SB	0.83 (0.73-0.95)*	Dropped	0.71 (0.52-0.98)*
Replace MVPA	1.16 (0.84-1.61)	1.40 (1.02-1.92)*	Dropped

CI = confidence interval; PR = prevalence ratio; MVPA = moderate-to-vigorous physical activity; SB = sedentary behavior. The PR was adjusted for sex, age, and marital status. * P < 0.05.

## DISCUSSION

This study examined the hypothetical effect of reallocation of time spent on active and sedentary activities on the prevalence of depressive symptoms in older adults. These results suggest that substituting sitting time for MVPA has positive effects on depressive symptoms. In addition, substituting sitting time with sleep resulted in benefits. These findings reinforce the benefits of these measures for preventing depressive symptoms, especially those related to an active lifestyle.^10^

A study on SB in older adults has also been highlighted. This age group spent the most time engaged in SB, as evidenced by the results of studies conducted in developed^30^ and developing^31^ countries. As a consequence, it has been reported that older people who spend more time in SB have worse health conditions^16^ and are at higher risk for depressive symptoms.^32^

Within a 24-hour period, SB comprises a significant portion of an individual’s time.^12^ Therefore, according to the present study, replacing 60 minutes/day of sitting time with MVPA or sleep can reduce the likelihood of depressive symptoms by 29 and 17%, respectively. Significant differences, albeit of lesser magnitude, were also observed when the amount of time replaced was shorter. Replacing activities with shorter durations may be more feasible for older adults.^33^

Recent studies have verified that replacing 30 minutes of time spent engaged in SB with the equivalent time in low-intensity physical activity and MVPA^22^ and transport time (walking/bicycling) and MVPA^23^ is also beneficial. Unlike the results of the present study, those presented by Yasunaga et al.^22^ showed no beneficial effects of the reallocation of SB time to MVPA in older Japanese adults. However, the authors found that replacing sedentary time with low-intensity physical activity resulted in decreased depressive symptoms. However, a study conducted by Wei et al.^23^ in older adults in the U.S. (National Health and Nutrition Examination Survey) showed that replacing SB with walking/bicycling or MVPA was associated with lower severity of depressive symptoms among older adults.

The practice of MVPA in older adults is considered a protective factor against adverse health conditions^34^ and mortality.^6^ Moreover, the relationship between MVPA and depressive symptoms has been verified both in cross-sectional studies^35^ ^,^ ^36^ and in longitudinal studies;^37^ ^,^ ^38^ thus, it can be considered a protective factor.

In addition, exercise programs reduce depressive symptoms. According to a systematic review and meta-analysis of 41 randomized controlled trials conducted with older adults, exercise is an effective treatment option for older adults with depressive symptoms.^39^

In addition to demonstrating that increased physical activity can reduce depressive symptoms, the results of the present study indicate that sleep plays a key role. Both long and short sleep durations are negatively associated with health^40^ ^,^ ^41^ and a higher risk of mortality in older adults.^42^ Furthermore, according to a meta-analysis of longitudinal studies, short and long sleep durations are risk factors for depressive symptoms.^43^

Among the studies that evaluated the effect of isotemporal substitution on depressive symptoms,^20^ ^,^ ^22^ the reallocation of sitting time was not tested with sleep. However, it has been shown that replacing 30 and 60 minutes of SB with an equivalent amount of time sleeping is associated with benefits in cardiovascular risk biomarkers^43^ and a lower mortality risk, respectively, in those who sleep for fewer than 7 hours.^19^

Thus, increasing the time spent sleeping seems to be a protective factor against depressive symptoms as sleep plays a role in homeostasis and body regulation, and its imbalance is associated with depression.^45^ ^,^ ^46^ According to a two-year follow-up study, older adults who sleep for fewer than 6 hours at night have a higher incidence of depressive symptoms than those who sleep for 7-8 hours.^47^

According to the consensus of a National Sleep Foundation expert panel, sleeping for fewer than 6 hours is associated with poorer health conditions, including physical and mental illnesses, compared with sleeping for 6-9 hours. The National Sleep Foundation further notes that sleeping for 9-10 hours or more is associated with diseases and an increased risk of mortality.^13^

Thus, it is noteworthy that the results supporting an increase in the time spent sleeping should be interpreted with caution, as duration and previous sleeping time should be taken into consideration in older adults. It is possible that the older adults in the present study who sleep for fewer than 6 hours would benefit from increased sleeping time.

Finally, the results of this study reinforce the need for additional evidence on interventions to reduce SB in older adults. A recent systematic review found that interventions for SB reduction appeared to be effective in the short-term in adults;^48^ however, evidence of this effect in older adults remains incipient.^16^

With the results presented in the study, health professionals and public policies should focus on the regular practice of PA, encouraging decreased time engaged in SB, reducing the risk of various health hazards, including depressive symptoms.

Some limitations of the present study should be considered, as it is a cross-sectional study, which does not allow the determination of cause-and-effect relationships, and subjective measures were used to measure the level of physical activity and SB. However, it is worth noting that the IPAQ instrument has been validated in Brazil and is widely used.^46^ A strong point of this study is the inclusion of sleeping time in the estimation of the hypothetical effect of isotemporal substitution and the different durations tested. This method is valuable and widely used in research, as it allows behavioral changes to be modeled, and can provide important insight for new studies and interventions without expending unnecessary time and resources on inefficient studies.

## CONCLUSIONS

Replacing SB time with the same amount of time spent sleeping or performing MVPA can lead to a reduction in depressive symptoms. The longer the reallocation time, the greater are the benefits.
